# Multibody Computer Model of the Entire Equine Forelimb Simulates Forces Causing Catastrophic Fractures of the Carpus during a Traditional Race

**DOI:** 10.3390/ani12060737

**Published:** 2022-03-16

**Authors:** Eleonora Pagliara, Alvise Pasinato, Alberto Valazza, Barbara Riccio, Federica Cantatore, Mara Terzini, Giovanni Putame, Annapaola Parrilli, Maria Sartori, Milena Fini, Elisabetta M. Zanetti, Andrea Bertuglia

**Affiliations:** 1 Dipartimento di Scienze Veterinarie, Università degli Studi di Torino, 10095 Grugliasco, Italy; eleonora.pagliara@unito.it (E.P.); alberto.valazza@unito.it (A.V.); barbararicciovet@gmail.com (B.R.); 2 Rossdales Equine Hospital, Newmarket, Suffolk CB8 7NN, UK; alvise.pasinato@gmail.com; 3 Pool House Equine Clinic, Fradley, Lichfield WS13 8RD, UK; fedecantatore10@gmail.com; 4 Department of Mechanical and Aerospace Engineering (DIMEAS), Politecnico di Torino, 10129 Torino, Italy; mara.terzini@polito.it (M.T.); giovanni.putame@polito.it (G.P.); 5 Center for X-ray Analytics, Empa—Swiss Federal Laboratories for Materials Science and Technology, 8600 Dübendorf, Switzerland; annapaola.parrilli@gmail.com; 6 IRCCS-Istituto Ortopedico Rizzoli, Complex Structure of Surgical Sciences and Technologies, 40136 Bologna, Italy; maria.sartori@ior.it (M.S.); milena.fini@ior.it (M.F.); 7 Dipartimento di Ingegneria, Università degli Studi di Perugia, 06123 Perugia, Italy; elisabetta.zanetti@unipg.it

**Keywords:** horse, Palios, catastrophic carpal fracture, kinematics, micro-computed tomography, inverse dynamic analysis, multibody dynamic simulation, in silico modelling, computed modelling, racehorse welfare

## Abstract

**Simple Summary:**

Palios are traditional horseraces held in the main square of few Italian cities. Due to peculiar features of such circuits, adapted to the square architecture and thus characterized by tight curves and unconventional footing surface, horses involved are at particular risk of accidents. Prevention of catastrophic musculoskeletal injuries is a significant issue and matter of debate during these events. In particular, the negotiation of the curves in the city circuits is a significative concern. An experiment was set up to build a model of entire forelimb at the point of failure in the context of a turn comparable to that in a Palio circuit. The model was informed by live data and the output compared to post-mortem findings obtained from a horse that sustained a catastrophic fracture of the carpus during this competition. The objective of this study is to determine the magnitude and distribution of internal forces generated across the carpus under which the catastrophic injury has occurred and describe related post-mortem findings.

**Abstract:**

A catastrophic fracture of the radial carpal bone experienced by a racehorse during a Palio race was analyzed. Computational modelling of the carpal joint at the point of failure informed by live data was generated using a multibody code for dynamics simulation. The circuit design in a turn, the speed of the animal and the surface characteristics were considered in the model. A macroscopic examination of the cartilage, micro-CT and histology were performed on the radio-carpal joint of the limb that sustained the fracture. The model predicted the points of contact forces generated at the level of the radio-carpal joint where the fracture occurred. Articular surfaces of the distal radius, together with the proximal articular surface of small carpal bones, exhibited diffuse wear lines, erosions of the articular cartilage and subchondral bone exposure. Even though the data in this study originated from a single fracture and further work will be required to validate this approach, this study highlights the potential correlation between elevated impact forces generated at the level of contact surfaces of the carpal joint during a turn and cartilage breakdown in the absence of pre-existing pathology. Computer modelling resulted in a useful tool to inversely calculate internal forces generated during specific conditions that cannot be reproduced in-vivo because of ethical concerns.

## 1. Introduction

Italian Palios are traditional races held in a few medieval cities once a year. Jockeys ride bareback at high speeds. The track is located in the main square of the city center and the racing surface is composed of a mixture of tuff clay and sand [1], and its composition categorized as dirt [2]. Racehorses run clockwise on a triangular or quadrangular track with a perimeter ranging from 300 to 450 m. Due to the racetrack design and geometry, turns in traditional races are characterized by an extremely narrow curve radius, ranging from 95° to 92° in Siena Palio for example [1]. Properly designed flat racing racecourses have a constant curve radius, with a gradual transition in the radius of the turns, with a curve radius of 85 and 200 m or a 180° turn [3], and optimal banking superelevation to reduce centripetal force on the horse’s body while negotiating curves. 

Despite organizational efforts to reduce potential causes of accidents, the characteristics of Palio tracks raise many issues about the safety of horses involved in these competitions and significant concerns exist in the public opinion. Although an elevated incidence of musculoskeletal catastrophic injuries during Palios is only anecdotally reported, there is a perception among veterinarians involved in these competitions that fractures of the carpus are overrepresented in comparison with flat racing [4].

Horses that race during Palios are likely to be exposed to biomechanical forces that are different from those measured during conventional races. It is impossible to experimentally reproduce the exact conditions of a Palio race since the racetrack location is set up once a year. Furthermore, performing a direct biomechanical analysis on horses during the competition is unfeasible due to the reticence of the stakeholders. 

Computational modelling represents a viable alternative to experimentation in the field of biomechanics. In particular, numerical multibody models have proven to be a valuable tool for addressing open issues related to the musculoskeletal system as well as improving the understanding of complex anatomical structures, such as those constituting diarthrodial joints [5,6,7]. By exploiting these models, internal forces that cannot be measured in vivo directly (e.g., intra-articular contact forces) can be estimated to enable the identification of detrimental biomechanical conditions and allow the prediction of injuries [8,9].

In this study, we developed a computer model of the carpal joint at the point of failure in a horse which sustained a catastrophic injury during a Palio competition. Motion was introduced in the model considering the specific condition of the city circuit where the horse was running during the accident. The aim of this study is to evaluate the magnitude and the location of contact forces generated at the level of the carpus at the point of failure and to compare this output with experimental observations. 

## 2. Materials and Methods

### 2.1. Input Data

Our model was based on a limb collected from an adult Thoroughbred (TB) racehorse (7 years old; weighting 480 kg), which was euthanized because of a catastrophic fracture sustained during a traditional race (Palio of Asti; edition 2015). The horse sustained an injury while racing on a curve with a radius of 28 meters (Square Vittorio Alfieri), where the horse keeled over suddenly on the left forelimb (LF).

After the fall, the horse could not bear weight on the left forelimb, and its distal part was mechanically instable. First aid was immediately provided by immobilization of the limb in a well-padded splint. In the hospital a comminuted fracture with complete radial carpal bone failure of the left carpus was diagnosed by radiographic evaluation. The owner declined any treatment, and the horse was humanely euthanatized. 

#### 2.1.1. CT Scan

The model was based on Computer Tomographic image data (General Electric Hi-Speed Fx; detector rows of 2.0 mm each; collimation, 120 kVp; tube charge, 130 mAs; and pitch, 1.0; 512 × 512 px). The limb of the horse was detached at the level of the proximal radius after obtaining the consent of the owner, and immediately frozen. The specimen was placed with the carpus in full extension on the CT table into a leg-supporting pad with the long axis of the limb parallel to the CT gantry. Contiguous 2.0 mm transverse slices of the region of interest (ROI) were acquired, from the diaphysis of the radius to the distal phalanx. The specimen was examined in a proximal-to-distal direction along the transverse, sagittal and coronal cutting planes, and the generated images were viewed using a bone window. Elaboration in 2D and volume rendering of the bony structures were performed using DICOM software (OsiriX Lite; Pixmeo SARL, Bernex, Switzerland) and evaluated by an experienced radiologist. 

#### 2.1.2. Micro-Computed Tomography (µCT) 

The radius and the cuboid carpal bones with the corresponding articular surfaces were carefully dissected and further stored at −20 °C. All specimens were identified with an ID number. Specimens were submitted to µCT scan (high-resolution µCT Skyscan 1176 Bruker, Belgium) at the Istituto Ortopedico Rizzoli (Bologna, Italy). During µCT acquisition a 65 kV voltage and a current of 385 µA were applied to the source, interposing a 1 mm thick aluminum filter. Specimens were rotated 180° following a 0.4° rotation steps. The scan images obtained (1336 × 3936 pixels) had a nominal resolution (pixel size) of 17.5 µm and were then reconstructed with the NRecon program (version 1.6.10.4, Bruker) to obtain 5911 micro-tomographic sections. As correction factors for the reconstructions, in addition to the specific alignment relative to each single scan, beam hardening correction, smoothing and ring artefact reduction were used. Specimens were examined along the transverse, sagittal and coronal plains. Further volume rendering elaboration was performed. Volumes of interest (VOI) in the radial epiphysis and in the corresponding cuboid carpal bones were identified for quantitative analyses. Parameters measured included bone volume fraction (BV/TV), trabecular thickness (Tb.Th), trabecular separation (Tb.Sp) and number (Tb.N), trabecular pattern factor (Tb.Pf), structure model index (SMI), degree of anisotropy (Da) and C.Th. Bone mineral density was not considered for the analysis because of the potential bias related to the freezing process. 

#### 2.1.3. In Vivo Kinematic Measurements

A 470 kg horse (TB, 5yo gelding) ridden by a jockey weighing 60 Kg was equipped with a gait analysis system (Movit G1, Captiks, Rome, ITA) consisting of 4 IMU (triaxial accelerometer and triaxial gyroscope) over both carpi in a track used for the Palio racing training. Acquisitions were performed in a curve with a radius comparable to the curve where the accident occurred (Figure S1). IMU sensors were set to a sampling rate of 200 Hz and the recordings were saved on internal memory. To measure the live velocity of the horse galloping on the curve, a drone was positioned at a height of 20 m. A bright colored passive marker was positioned on the jockey’s hat and custom-made markers (circles of 50 cm diameter) were positioned on the ground at 5 m intervals along the outside of the curve. Two more cameras were positioned to make video footage of the horse from the side and from rear. All the videos were synchronized with IMU recordings. The horse reached maximum-speed gallop (13 m/s) over the 370 m diameter track in a clockwise direction. The recording session was kept as short as possible to ensure genuine IMU high-speed data without risk of miscalibration. Videos were digitized and analyzed with gait analysis software (Biomovie, ERGO55 version, Infolabmedia, Gressan (AO), Italy). Kinematic data derived from the experiment (stance duration, swing duration, stride duration, carpus angle at the impact, angle at max flexion, range of motion of the carpus) were imported in the model starting from live data. Distance (m), instant velocity (m/s), average velocity (m/s) and acceleration (m/s^2^) for 50 m of the curve at each lap on the video from above were calculated. The inclination angle (in degrees) between the metacarpus and the ground in posterior view was calculated from the video using the same gait analysis software. 

### 2.2. Computation Modelling

The CT scans images were segmented, and the respective 3D solid geometries of the entire forelimb were created using commercial software for 3D image processing (v.21.0, Mimics, Materialise, Leuven, Belgium). Specifically, since the radial carpal bone (Cr) was fractured into two main pieces, it was virtually reconstructed by aligning the bone fragments and applying a wrapping function to reproduce the non-fractured bone structure. Thus, the obtained 3D geometries were imported into multibody software (v.2017, MD Adams, MSC Software Corporation GmbH, Munich, Germany), where a dynamic model was implemented. The model included the following bones obtained from the CT scans: the radius, the Cr, the intermediate carpal bone (Ci), the ulnar carpal bones (Cu), the second (C2), third (C3) and fourth (C4) carpal bones, and the proximal metacarpal bone. In addition to the mentioned bones, the humerus, the ulna, the distal metacarpal bone, and the phalanges were included by adapting standardized geometries to the specific horse size. A value of density equal to 1590 kg/m^3^ was assigned to the bones based on published data [10]. Thus, the inertial characteristics and center of mass were computed for each body segment. Moreover, the body of the horse was simplified by using a cylindrical geometry having a mass equal to the body weight, that is, approximately 500 kg. 

**Figure 1:**
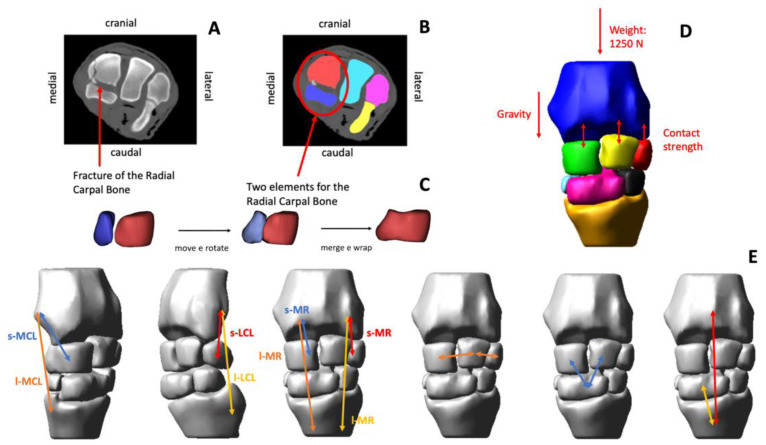
Workflow used to build a simplified equine carpus model (**A**–**E**). The radial carpal bone (Cr) was fractured into two main pieces (**A**). The fractured volumes of the Cr were segmented (**B**). The Cr was virtually reconstructed by aligning the bone fragments and applying a wrapping function to reproduce the non-fractured bone surface (**C**). All the articulating bones of the carpal joint were segmented, and the respective three-dimensional (3D) solid geometries were created using software for 3D image processing. The 3D model was imported in multibody software and the gravity, and the weight forces were introduced (**D**). The collateral ligaments and the dorsal retinaculum were modelled (**E**). The short and long medial collateral ligaments (s-MCL and l-MCL), and the short and long lateral collateral ligaments (s-LCL and l-LCL) were introduced in the model. The short and long medial retinaculum (s-MR and l-MR) blended with the dorsal joint capsule and fascia were modelled. The intercarpal ligaments were modelled as bushing elements, acting between two bones proportionally to their relative linear and angular displacements and velocities.

The elbow, carpus, fetlock, and distal interphalangeal joint were modelled as hinges, thus, each of them allowed for only a rotation movement (i.e., 1 degree of freedom) on the sagittal anatomical plane, whereas the articulations between proximal and middle phalanges were considered fixed. Since this study was focused on the investigation of the forces generated inside the carpal joints, contacts between the articulating surfaces of the carpal bones and the retaining actions of the articular ligaments were implemented. The complexity of the joint, due to the high number of its anatomical structures (i.e., the various osseous segments, ligaments, and tendinous structures), required the introduction of some modelling simplifications. First, the relative motion among the C2, C3 and C4 bones was neglected by fixing them together. Secondly, only the collateral ligaments, the dorsal retinaculum and the palmar carpal ligament were modelled whereas the constraining effect of the intercarpal ligaments and soft tissues that surround the joint was represented by means of bushing elements which work as a “soft volume” with assigned stiffness properties and viscosity. Specifically, each bushing element was implemented to generate a six-component force (i.e., three translational forces and three torques) acting between two bones proportionally to their relative linear and angular displacements and velocities (Appendix A, Bushing element implementation). Moreover, each collateral ligament was represented by means of two branches, i.e., the long and short medial collateral ligament (l-MCL and s-MCL), and the long and short lateral collateral ligament (l-LCL and s-LCL). The dorsal retinaculum was represented by two branches per joint side, i.e., the long and short medial retinaculum (l-MR and s-MR) and the long and short lateral retinaculum (l-LR and s-LR). The action of the palmar carpal ligament was considered to counterbalance the stabilization effect offered by the digital flexor tendons [11]. Each branch was modelled as a non-linear tension-only spring [12] having the characteristic stiffness parameters adapted from the literature and the slack lengths of the ligaments tuned, by means of preliminary simulations, starting from the modelled ligament lengths measured with the joint at its resting position. Origin and insertion points of the branches were obtained from anatomical references [13,14]. Visual details of the model are reported in Figure 1 and details of mathematical functions are described in Appendix A.

The intra-articular contact forces were calculated by means of an interpenetration function taking into account the total cartilage thickness (C.Th) between the contacting bones derived from the µCT scan. Three different contact pairs were defined between the distal radius and the Cr, between the distal radius and the Ci, and between the distal radius and the Cu bones, respectively. Articular contacts were assumed to be frictionless. In addition, a further contact was defined between the limb and the ground. Further, the gravity force was considered for the simulation. Lead and trail limb stride duration (s), stance duration (s), swing duration (s), carpus angle at impact (deg), angle of max extension (deg), angle of maximal flexion (deg) and range of motion (deg) were imported in the model in conjunction with gyroscope and accelerometer raw data captured by IMU sensors. 

**Figure 2:**
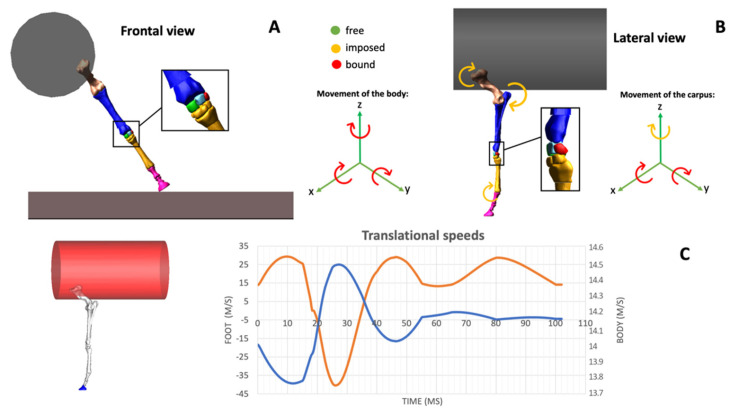
Introduction of kinematics data in the simplified equine carpal model (**A**–**C**). The body of the horse was simplified by using a cylindrical geometry (in grey) having a mass equal to the body weight. The frontal view of the forelimb which sustained the injury and the corresponding inclination with respect to the ground were introduced in the simulation and visualized during the stance phase of the stride (**A**). The animation is shown in Video S1. The position of the same limb during the stance phase of the stride was visualized in the lateral view (**B**). The translational speeds of the body of the horse and the foot, derived from data captured using inertial measurement units, are visualized in the corresponding graph (**C**). On the right part of the graph the speed of the body is scaled, on the left part the speed of the foot is scaled. The blue line joins the value of 0 at 18 ms from the beginning of the simulation; this corresponds to the moment when the foot hits the ground.

Furthermore, video footage of the race, acquired from different points of view, was analyzed to extract useful information to replicate the dynamic conditions of the accident. As the maximum speed reached by the horse along the curve was calculated as 14 m/s, kinematics measurements were properly scaled in time and referred to the joint. The inclination of the affected limb metacarpus to the ground was calculated as 56°. The video frame taken into consideration for the calculation of the angle was the frame where the maximal metacarpal inclination was reached at the end of the curve. The whole simulated stride corresponded to 0.102 ms. Kinematic data introduced in the model are shown in Figure 2. 

### 2.3. Pathology

#### 2.3.1. Macroscopic Evaluation of the Specimen

**Figure 3:**
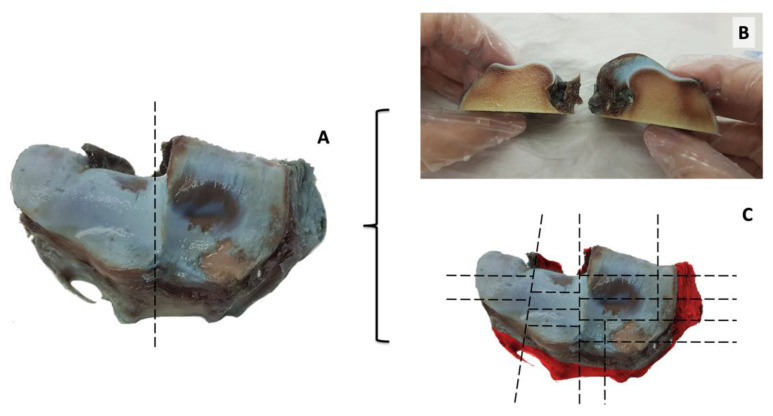
Macroscopic aspect of the articular surface of the distal radius. The articular surface is visualized after dissection of the radio-carpal joint, and Indian ink deposition. Hyaline articular cartilage is severely damaged, as shown by extensive erosion on the radial facet (**A**). A first cut was performed along the dotted line presented in A (**B**). Multiple cutlines are planned in the articular surface of the radio-carpal joint to capture lesions in the entire hyaline articular cartilage surface at histopathology (**C**).

Macroscopic evaluation of the left carpal joints of the euthanatized horses was performed after careful dissection and disarticulation of the joint compartments and scored accordingly [8]. Indian ink solution diluted with phosphate-buffered saline (1:5) was used to stain the superficial hyaline cartilage as described in [15] and the stained specimens were photographed using a high-resolution digital camera (Figure 3). Findings were evaluated according to the classification reported in the Table 1.

#### 2.3.2. Histopathology

After µCT assessment, specimens were stored again in phosphate-buffered saline at +4 °C. Specimens were processed for histological analysis. Multiple cutlines were made in the articular surface of the distal radius to map the entire articular cartilage of this specimen in relation of the severity of the macroscopic lesions (Figure 3B,C). 

The cuboid carpal bone was not processed for histopathology. Fourteen osteochondral pieces were obtained. Cuts for histological examination were preferentially performed in the regions where macroscopic cartilage lesions were observed. After fixation in 10% formalin for 24h and rinsing in water, specimens were decalcified with a 5% formic acid solution and 4% hydrochloric acid in 1 L distilled water, respecting the proportion for the desired volume, and remained in a descaling solution for one month, where the hardness of the bone component was systematically checked. After descaling, samples were rinsed in water and then dehydrated in an increasing series of ethyl alcohol (70%, 95% and 100%), passed in xylol and included in paraffin blocks. For each osteochondral piece, two sections of 5 µm thickness were cut along the transverse plane of the carpus to observe both the hyaline cartilage and the corresponding subchondral bone (SCB). Sections were stained with hematoxylin and eosin and with safranin O fast green. The stained sections were then acquired at 100x for further analysis through a digital scanner (Leica Biosystems) and assessed by one author using the OARSI osteoarthritis cartilage histopathology assessment system [15,16] for grading the articular cartilage damage. A site-by-site comparison was performed between the histopathology evaluation of the hyaline cartilage and the corresponding µCT sections at the level of the SBC. Cartilage scores of each section are reported and described in Table 2.

## 3. Results

### 3.1. Radiology and CT Scan

Radiographic examination of the carpus identified a biarticular and moderately displaced fracture of the Cr. The CT scan confirmed a complete fracture along the coronal plane of the Cr, propagating from the radio-carpal to the inter-carpal joints, with comminution of the palmaro-proximal aspect of the Ci. The main fracture line divided the Cr in two main pieces, with the dorsal portion approximately two thirds and the palmar approximately one third of the whole bone (Video S2).

### 3.2. Computation Modelling

The kinematic input in the model was derived from the experimental acquisitions in the training racetrack and measurement on video footage. 

**Figure 4:**
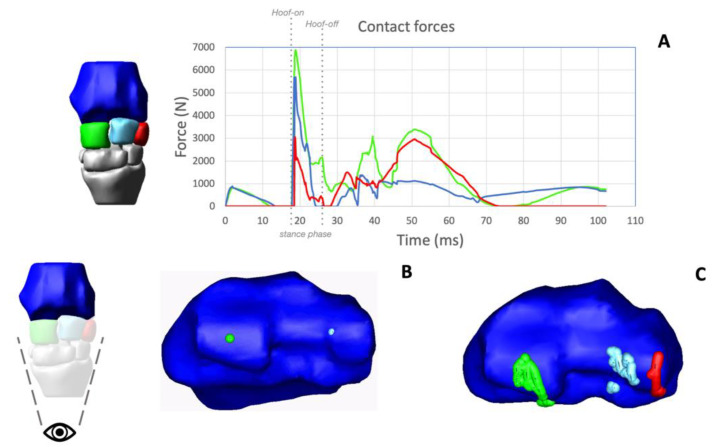
Outputs of the model. The simulated stride shown in the output is 103 ms long. The plot shows the magnitude of the forces of contact between the radius (Ra) and the radial carpal bone (green), the intermediate carpal bone (light blue) and the ulnar carpal bone (red). The magnitude of the forces is reported in Newtons as a function of the time of the simulated stride. The hoof-on/hoof-off points and the duration of the stance phase are included in the graph (**A**). The articular surface of the 3D model of the distal Ra (articular surface of the radio-carpal joint) is shown in (**B**). The video animation (Video S3) shows the migration pattern of the center of contacts (CoCs) along the simulated stride. The green dots define the CoC between the Ra and radial carpal bone, the light blue dots define the CoC between the Ra and intermediate carpal bone, and the red dots define the CoC between the Ra and ulnar carpal bone. Tracking position of the contact forces are shown in (**C**).

All the proximal articular surfaces of the Cr, Ci and Cu resulted in a contact with the articular surface of the radius after 0.18 ms from the beginning of the simulated stride (Figure 4A). 

Locations and magnitudes of the center of contact (CoC) forces at the level of the radio-carpal joint were obtained as output from the computational simulation. Contact forces presented a peak soon after the limb hit the ground, and maximal values differed among the three cuboid bones. The peak force between the radius and Cr had the higher value (around 7000 N), and the peak force between the radius and Cu had the lowest value. When the limb started the swing phase, contact forces decreased, to increase again during full flexion of the radio-carpal joint, but without reaching similar values to those obtained during the contact with the ground. The localizations of the CoC between the Cr-radius, the Ci-radius and the Cu-radius, obtained by tracking the CoC in the corresponding articular facets of the radius, were visualized (Figure 4B,C). CoCs were localized on the convexity of the radial trochlea at the beginning of the simulation, when the carpus was in complete extension and then moved, at approximately 0.40 msec from the start of the simulation, on the palmar aspect of the articular surface of the radius, during the flexion of the joint, and then returned to the dorsal aspect of the articular surface of the radius during carpal extension. 

### 3.3. Pathology

The articular surfaces of the radial and intermediate facets of the radius, medially and laterally to the medial parasagittal groove (MPG), and the ulnar facet of the radius exhibited diffuse wear lines, and two large patchy areas with an articular cartilage erosion. The Cu presented multiple wear lines on the articular surface, whereas the articular surfaces of the Cr and Ci exhibited irregular and full thickness cartilage erosions. The articular surface of the radio-carpal joint was classified as having severe cartilage damage, due to the extensive retention of Indian ink at the level of the hyaline cartilage. The direction of the wear lines corresponded with the physiologic rotation axis of the joint. 

#### 3.3.1. Qualitative and Quantitative µCT Analysis

**Figure 5:**
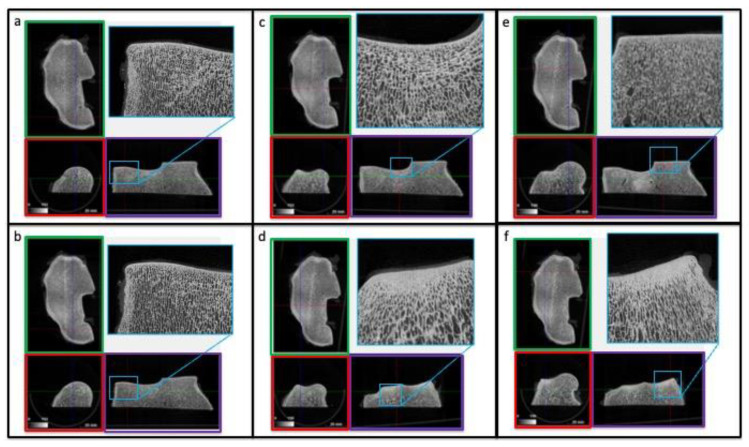
Qualitative µCT analysis of the distal radius (radio-carpal joint). Panels (**a**–**f**) represent different regions of the radial epiphysis. Moderate bone densification is limited over the subchondral bone regions, at the level of the dorsal aspect of the radial facet of the radius (**d**,**f**). The sagittal (red box), coronal (green box) and transverse planes (violet box) of the distal radial epiphysis are shown. The plane of cuts of the µCT are indicated within the corresponding color borders.

The µCT dataset allows the observation of different anatomical planes and the analysis of the trabecular pattern of the SCB of the distal radius and cuboid carpal bones. We used both Dataviewer (Bruker MicroCT, Kontich, Belgium) and Image J software loading the entire datasets as virtual stacks. Observing the articular surface of the radius using the false color light blue attributed to the hyaline cartilage, two regions with focal cartilage damage were detected at the level of the radial facet of the radius, accordingly with macroscopic observation (Video S4). Lesions were localized medially to the medial parasagittal ridge of the radius (MPR), on the dorsomedial border of the bone and in the palmar aspect of the radial trochlea. Further cartilage lesions were observed in the intermediate facet of the radius laterally to the MPR. In the SBC, neither focal porosities nor surface irregularities were detected. Sections along the sagittal plane revealed only a moderate increase in bone density in a limited area over the SCB regions at the level of the dorsal aspect of the radial facet of the radius, and on the dorsal aspect of the Cr and Ci (Figure 5). According to the current literature, areas of sclerosis in these locations reflect adaptation of the bone to repetitive loads, so in the case of this seven-year-old TB racehorse with a long training and racing career they were considered likely secondary to chronic modelling. Three volumes of interest (VOIs) of 15 mm in size were selected on the articular surface of the radio-carpal joint, based on the severity of deterioration of the articular cartilage. VOI 1 and 2 were localized medially to the MPR: VOI 1 was opposite the fracture line on the Cr. VOI 3 was a negative control and was located abaxially to the MPR, at the level of the intermediate facet of the radius. The VOIs sampled in the distal articular surface of the radius showed marked differences in cartilage thickness (C.Th): in the area identified with VOI 1, it was detected that the minimum value of C.Th was 76.78 µms. All structural µCT parameters were reported in detail in the corresponding table (Table 3).

#### 3.3.2. Histopathology

**Figure 6:**
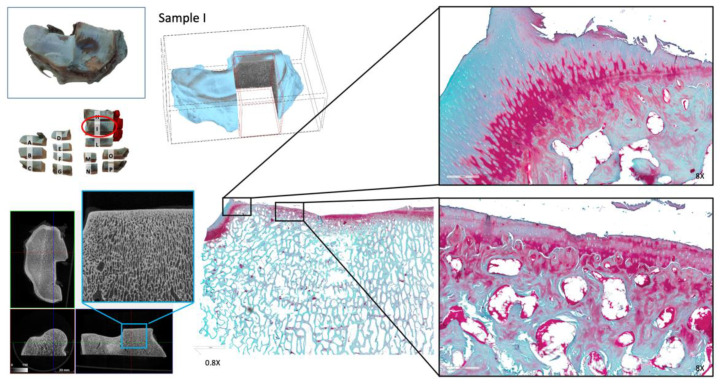
Histopathology specimens of the hyaline cartilage and subchondral bone of the articular surface of the radius (radio-carpal joint). The radial facet of the articular surface of the distal radius is shown. Close-up details of the histopathology of the hyaline cartilage of the radial facet are displayed. The image captured the radial facet, with extensive destructive changes to the hyaline cartilage.

Sections stained with safranin O fast green (SOFG) were assessed using a validated grading system (Table 2). The sections stained with hematoxylin and eosin (H&E) were assessed for comparison. Hyaline cartilage at the level of the radial facet of the radius appeared extensively damaged (Figure 6). 

## 4. Discussion

In this study, a multibody computer model of the entire equine forelimb was generated to derive the magnitude and the localization of the contact forces at the level of the radio-carpal joint in a horse experiencing a catastrophic carpal fracture during a traditional race. This point-of-failure computer model was informed by data that we obtained from the literature and from kinematic data taken from a live test that reproduced, as closely as possible, the conditions under which the injury occurred. 

Recently, a musculoskeletal model of the Thoroughbred forelimb was developed to test strain profiles of the tendons and ligaments at trot and canter [17] and the same model was used to study the response related to a changing ground surface [17]. In contrast to that model, where bones were simulated by rigid elements, we developed a model of the carpal joint where biomechanical properties of tissues (cartilage, trabecular bone, ligaments) at the level of the carpus and their deformation were considered. Computation modelling using a finite element method has been previously applied to the distal portion of the third metacarpal bone and proximal phalanx in racehorses to study deformation of the trabecular bone and gain information on stress fracture pathogenesis [18,19,20]. Finite element simulation is informative of the stress and strain properties of the material, whereas a multibody system is useful to capture the interaction between two different surfaces and study contact forces. The proposed approach is useful to study the interaction between articular surfaces during motion and describe forces at the instant preceding failure, introducing specific conditions of speed and inclination with respect to the ground. 

The introduction of live kinematic data captured in simulated conditions adds value to the output of the model in relation to the specific injury under study. Further validation of the model itself would be recommended before using this model to describe different conditions. The peak of the contact forces in our simulation was identified at the level of the cartilage surface of the radial facet of the distal radius, axially to the medial parasagittal ridge, and occurred 18 ms from the beginning of the stance phase of the stride in the simulated period. We assumed that these findings were explained by the consistent inclination of the leading forelimb and the consequent displacement of the center of mass of the horse’s body negotiating the curve at elevated speed. The peak of the forces, based on the reconstruction of the entire horse’s stride in this specific race circuit, is localized in correspondence to the articular surface where the fracture line would probably start. It is important to note that the amount of contact force values predicted by our model do not provide a direct explanation of the fracture per se. We estimated a contact peak force close to 7000 N at a single point between the radial facet of the radius and the Cr. This value is only indicative, because the absence of the forces attributed the flexor and extensor tendons in our model underestimates the force acting on the carpus in this horse. However, values obtained in the model are in line with those reported in the equine carpal joints in previous studies [18,21].

There are limited data on the fatigue properties of carpal bone due to the technical difficulties encountered in vivo to measure such loads without altering the physiological loading conditions [22]. Currently, there are not any precise references to the amount of force that a carpal bone can withstand before failure. The study by Tidswell [23] shows that an increase in bone mineral density is a common finding in equine cuboidal bones, leading to a greater stiffness in racehorses under training. However, no study can precisely define the cuboidal bone’s breaking point. 

In the literature it is reported that the curve in a circuit is a significative factor in the pathogenesis of racehorses’ injuries [3,24,25]. Kinematics of movement on a curved track has been recently investigated in horses at trot [26] and at gallop [27] on racetracks where the radius of the curve ranged from 85 to 200 m. In horses galloping on a curve at around 14 m/s, the entire limb and the third metacarpal bone inclination increase, to counterbalance the elevated centripetal acceleration acting perpendicular to the direction of motion [3,27]. At the extremal point of a curve, a horse generally experiences forces that are smaller than what would be predicted by mathematical models [28]. It has been suggested that increased force associated with galloping on a curve may be associated with increased injury risk [25,29]. Commonly, horses at gallop enter curves with the leading limb on the inside of the curve; however, the horse in the current study had the leading limb, which was the one that sustained the fracture, on the outside of the curve. It is possible that this choice could be the result of the horse’s preference [30] or it could be led by the lack of specific training to race on such a small track. 

The simulation of the migration of the forces during an entire stride in our model represents a useful indication of the potential detrimental effect of the force concentration on the proximal aspect of the cuboid bones of the carpus when the horse is leaning on a curve. The pattern of migration of the center of contacts of the forces in that joint matches with the observed pattern of hyaline cartilage injuries observed in the horse under study. The maximum load experienced by the radial carpal bone peaked when the carpal joint was in the closed position, at the beginning of the stance phase, and the load reached a point where bone failure could have been possible. Furthermore, as the response of the trabecular bone to repetitive loading is anisotropic, the resistance of the bone is higher when the cyclic loading is applied in the direction of the physiological loading angle and lower at other loading angles [31]. In cases of extreme leaning, such as in the conditions under study, the specific load is applied in a non-axial direction so the resistance of the bone can be further decreased. 

Complete fractures of the radial carpal bone are uncommon in racehorses in flat racing [32]. It seems logical to correlate the unusual design of the circuit in the traditional race, mainly the small radius of the curves (28.4 m) of the track travelled at such high speed, with the observed injury. In the specific context of Palio, a horse is not regularly trained in these conditions as the circuit is built once a year, so we have concerns about the ability to develop bone adaptation necessary to this specific race.

At the entry of the curve considered in our modelling, the carpal joint of the forelimb negotiating the curve outwardly is overwhelmed by the elevated centripetal acceleration [27] due to the displacement of the center of mass of the animal, with a tremendous increase in the contact forces in the medial aspect of the radio-carpal joint. 

The identification of a severe cartilage breakdown on the radial facets of the distal radius requires some specific considerations concerning data introduced in the model. We must consider that catastrophic fractures in horses could be the consequence of pre-existing osteoarthritis of the carpus, as a risk factor for the fracture. However, histopathology and microstructural analysis on the specimen under study did not support pre-existing lesions in this horse. The lesions observed in the hyaline cartilage appeared acute rather than related to chronic injury. The cartilage damages were not uniformly distributed, and interestingly, they were localized with good approximation to the points where the model predicted the higher contact in the joint. We observed no histological evidence of chronicity of the lesions, such as a chondrocyte cluster or a reduction in chondrocyte density.

Micro-CT revealed increased bone density only in very small areas of the joint, such as the dorsal aspect of the intermediate carpal bone and on the radial facet of the distal radius, as we expected in a racehorse, and reflecting bone physiological adaptation to training [23,33]. Based on current knowledge, the fracture of the radial carpal bone in this horse was generated under compression, due to the high loading rate [34,35,36]. Bone geometry, material properties, load direction and the speed of application of the force are the main factors involved in this failure [37]. Furthermore, the cuboid bones in the lateral aspect of the carpus allow greater dissipation of the axial loading thanks to the arrangement of the fourth carpal bone between the ulnar and the intermediate carpal bone, due to the horizontal translation of the bones and their recoil, and the strong intercarpal ligaments [38,39]. This type of mechanism is not possible on the medial aspect of the joint.

The proposed model highlighted some critical elements that could lead to acute bone failure during traditional races. This is only a pilot study, and more work is required to validate this model. The future goal will be to examine more cases of catastrophic fractures to identify specific forces generated in this type of circuit and compare data derived from the simulation with the yield points of equine carpal bones. These types of models, such as the one used in this study, which integrated in vivo kinematics within silico modelling, could be useful for designing racetracks to simulate the potential risks for horses competing on these courses. In terms of prevention of this type of injury, this study supports the consideration that city tracks should be redesigned to increase the safety of turns and to accommodate horses running at high speed.

Our model presents some limitations: first of all, not all the sites where macroscopic lesions were observed were completely predicted by the model. This is probably due to the necessary simplifications introduced in the model. The palmar contention performed by the flexor tendons was not fully considered in the model. The model was retrospectively created and refers to a single horse. Therefore, the findings reported by this study cannot be translated to the whole population of racehorses competing in traditional races. In addition, it is not possible to experimentally simulate the track conditions where the accident occurred for obvious ethical reasons.

## 5. Conclusions

Computer simulation at the point of failure can define with precision the internal forces exceeding the yield point of the bone and cartilage and causing catastrophic fracture of the carpus in a horse galloping during a traditional competition. This study describes a new approach to investigating fractures in racehorses, requiring further validation studies. The model is simpler than finite element simulation and could be applied to specific fractures, introducing proper input. This approach has the potential to be applied to fractures other than the one studied and offers the opportunity to simulate new racetrack design to improve racehorses’ welfare and hopefully reduce catastrophic accidents.

## Figures and Tables

**Table 1:** Classification of cartilage lesions based on macroscopic evaluation after Indian ink staining.

Grade 1	Intact surface (Surfaces are normal and smooth in appearance and did not retain Indian ink)
Grade 2	Minimal fibrillation (Surface retains Indian ink as elongated specks or light-grey patches)
Grade 3	Overt fibrillation (Area were velvety in appearance and retains Indian ink as intense black patches)
Grade 4	Erosion (Area of cartilage exposing the underlying subchondral bone)

**Table 2:** Histopathology grading of the hyaline cartilage of the entire surface of the distal radius, according to a modified Mankin score [8].

Grade 0 Intact cartilage surface and intact cartilage morphology
Grade 1 Surface fibrillation
Grade 2 Surface discontinuity
Grade 3 Wear lines in the cartilage
Grade 4 Cartilage erosion
Grade 5 Cartilage denudation

**Table 3:** Quantitative µCT data of selected volume of interest (VOI) from the antebrachio-carpal joint, taken at the level of the distal radius, as shown in the corresponding illustration. Parameters measured included bone volume fraction (BV/TV), trabecular thickness (Tb.Th), trabecular spacing (Tb.Sp) and number (Tb.N), fragmentation index (Tb.Pf), structure model index (SMI), degree of anisotropy (Da) and cartilage thickness (C.Th). The C.Th. has been reported as a mean value, and with the minimal and maximal value.

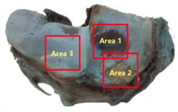	**Trabecular Bone**								**Cartilage**
	VOI	BV/TV	Tb.Th	Tb.SP	Tb.N	Tb.Pf	SMI	Da	C.th
		%	μm	μm					μm (min-max)
	Total	40.76	309.44	507.32	1.32	−0.19	−0.09	0.54	
	Area 1	35.4	199.89	514.88	1.77	−0.44	0.15	0.32	224.59 (76.78–579.90)
	Area 2	51.52	334.81	423.31	1.54	−2.44	−1.37	0.4	312.29 (99.61–567.29)
	Area 3	41.21	273.97	515.78	1.5	-0.73	-0.35	0.56	629.83 (319.86–825.79)

## Data Availability

Not applicable.

## Supplementary Materials

The following supporting information can be downloaded at: https://www.mdpi.com/article/10.3390/ani12060737/s1, Figure S1: View from above and planimetry of the surrogate track used to simulate in vivo kinematic measurement during turn; Video S1: Simulation of an entire stride reconstructed along 110 msec. Video S2: Macroscopic aspect of the fracture lines was elaborated in the corresponding CT scans of the carpus. Video S3: The migration pattern of the center of contacts (CoCs) along the simulated stride. Video S4: Elaborations in 3D and volume rendering of the distal radial epiphysis. False colors were attributed to tissues in relation to their structural density, using yellow ochre for the bone and light blue for the cartilage.

## Appendix A

### Appendix A.1. Mathematical Detail of the Model

#### Spring Element Implementation

The force–strain relationship of each spring element is described by the following non-linear piecewise function:

$$f=\{\begin{array}{cc} -k\left(\varepsilon -\varepsilon_{L}\right), & \;\;\varepsilon >2\varepsilon_{L}\\ -0.25\;k\frac{\varepsilon^{2}}{\varepsilon_{L}}, & \;\;0\leq \varepsilon \leq 2\varepsilon_{L}\\ 0, & \;\;\varepsilon <0 \end{array}$$

where *ε* is the ligament strain, *ε_L_* is a reference value of the strain assumed to be 0.03 and *k* is the stiffness parameter, expressed as force per unit strain, of each different spring element. The spring strain *ε* is defined as:

$$\varepsilon =\frac{\left(l-l_{0}\right)}{l_{0}}$$

where *l* is the actual element length and *l_0_* is the no load length (i.e., slack length). Moreover, to avoid high frequency vibrations during simulations, a parallel damper with a damping coefficient of 0.5 Ns/mm was added to each spring element.

**Table A1:** Characteristic parameters of the spring elements.

Element	k (N)	l_o_ (mm)
l-MCL	48,000	102.0
l-LCL	48,000	105.9
l-MR	10,000	106.9
l-LR	10,000	107.2
s-MCL	24,000	51.4
s-LCL	24,000	50.0
s-MR	1000	53.0
s-LR	1000	42.4

### Appendix A.2. Bushing Element Implementation

The force–displacement relationship of each bushing element is described as follows:

$$\left[\begin{array}{c} F_{x}\\ F_{y}\\ F_{z}\\ T_{x}\\ T_{y}\\ T_{z} \end{array}\right]=\left[\begin{array}{cccccc} K_{11} & 0 & 0 & 0 & 0 & 0\\ & K_{22} & 0 & 0 & 0 & 0\\ & & K_{33} & 0 & 0 & 0\\ & & & K_{44} & 0 & 0\\ & sym & & & K_{55} & 0\\ & & & & & K_{66} \end{array}\right]\cdot \left[\begin{array}{c} x\\ y\\ z\\ \alpha \\ \beta \\ \gamma \end{array}\right]+\left[\begin{array}{cccccc} C_{11} & 0 & 0 & 0 & 0 & 0\\ & C_{22} & 0 & 0 & 0 & 0\\ & & C_{33} & 0 & 0 & 0\\ & & & C_{44} & 0 & 0\\ & sym & & & C_{55} & 0\\ & & & & & C_{66} \end{array}\right]\cdot \left[\begin{array}{c} V_{x}\\ V_{y}\\ V_{z}\\ W_{\alpha }\\ W_{\beta }\\ W_{\gamma } \end{array}\right]$$

where *F* and *T* are the translational and torsional force components, respectively; *x, y* and *z* are the relative translational displacements between connected bodies; *α, β* and *γ* are the relative rotational displacements between connected bodies; *K*_11_*, K*_22_ and *K*_33_ are the translational stiffness coefficients; *K*_44_*, K*_55_ and *K*_66_ are the rotational stiffness coefficients; *Vx, Vy* and *Vz* are the relative translational velocities between connected bodies; *Wα, Wβ* and *Wγ* the relative rotational velocities between connected bodies; *C*_11_*, C*_22_ and *C*_33_ are the translational damping coefficients; *C*_44_*, C*_55_ and *C*_66_ are the rotational damping coefficients.

**Table A2:** Characteristic parameters of the defined bushing elements.

Connected Bodies	K_11_ (N/mm)	K_22_ (N/mm)	K_33_ (N/mm)	K_44_ (N/°)	K_55_ (N/°)	K_66_ (N/°)	C_11_ (Ns/mm)	C_22_ (Ns/mm)	C_33_ (Ns/mm)	C_44_ (Ns/°)	C_55_ (Ns/°)	C_66_ (Ns/°)
Metacarpus—Radius #1 *	1	1	1	10	10	10	0	0	0	0	0	0
Metacarpus—Radius #2 *	0	0	0	5	5	5	0	0	0	0	0	0
Metacarpus—Radius #3 *	0	0	0	50	50	50	0	0	0	0	0	0
C3—Cu	3000	1000	3000	15	15	15	3000	3000	500	15	15	15
C3—Ci	500	500	500	4	4	4	3000	3000	500	15	15	15
C3—Cr	1000	1000	500	5	5	5	3000	3000	500	15	15	15
C3—Metacarpus	1500	1500	1500	15	15	15	3000	3000	3000	15	15	15
Ci—Cu	0	0	0	0	0	0	0	0	2000	0	0	0
Ci—Cr	0	0	0	0	0	0	0	0	2000	1	1	1

* Three alternative bushing elements were used for the Metacarpus—Radius coupling. Such bushings were activated sequentially during the simulation. In detail, bushing #1 was activated for the 0–26 ms time interval, #2 for the 26–46 ms time interval, and #3 for the 46–102 ms time interval.

### Appendix A.3. Contact Implementation

Contact forces *F_c_* were calculated by using the following interpenetration formulation:

$$F_{c}=K_{c}\delta^{e}+C_{c}\left(\delta ,\;\delta_{max}\;,C_{max}\right)\dot{\delta }$$

where *K_c_* is the contact stiffness constant, *δ* is the interpenetration depth between contacting rigid bodies, *e* is a nonlinear power exponent, $\dot{\delta }$ is the penetration velocity and *C_c_* is a damping function defined as:

$$C_{c}\left(\delta \right)=\{\begin{array}{cc} 0, & \delta \leq 0\\ C_{max}{\left(\frac{\delta }{\delta_{max}-\delta }\right)}^{2}\left(3-\frac{2\delta }{\delta_{max}-\delta }\right), & 0<\delta \leq \delta_{max}\\ C_{max}, & \delta \geq \delta_{max} \end{array}$$

where $\delta_{max}$ is the maximum interpenetration depth, defined equal to 0.1 mm, at which the damping function reaches its maximum value $C_{max}$ of 30 Ns/mm, which was adapted from data reported for the human knee.

Specifically, the parameter *K_c_* was computed by combining the following formulas:

$$E=\frac{H\left(1+\nu \right)\left(1-2\nu \right)}{1-\nu }\;K_{c}=E\frac{A}{s}$$

where *E* is the Young’s modulus, *H* is the aggregate modulus, *ν* is the Poisson’s ratio and *s* is the thickness relative to the cartilage layers of the contacting bones. Moreover, the contacting area *A* between bones was initially estimated from the CT scans.

The nonlinear power exponent was iteratively refined so that a maximum reported contact force equal to 15,000 N could be reached for the maximum considered interpenetration depth, that is, the total cartilage thickness.

Based on the same formulation, a further contact was defined between the ground and the front limb. In this case, friction was considered by using a Coulomb’s model. Due to the lack of data in the literature, generic contact parameters were iteratively adapted to the case study.

**Table A3:** Parameters involved in the articular contact.

Contact Pair	Aggregate Modulus *H* (MPa)	Young’s Modulus *E* (MPa)	Poisson’s Ratio *ν*	Area *A* (mm^2^)	Cartilage Thickness *s* (mm)	Max Damping *C_max_* (Ns/mm)	Max Penetration *δ_max_* (mm)	Power Exponent *e*	Stiffness *K_c_* (N/mm)
Radius—Cr	1.58	1.38	0.22	958.7	2.23	30	0.1	8.4	955.6
Radius—Ci	1.42	1.29	0.19	845.9	2.16	30	0.1	11.65	855.8
Radius—Cu	1.62	1.34	0.26	347.8	2.20	30	0.1	12.35	343.2

**Table A4:** Parameters involved in the ground–limb contact.

Contact Pair	Max Damping *C_max_* (Ns/mm)	Max Penetration *δ_max_* (mm)	Power Exponent *e*	Static Friction Coefficient	Dynamic Friction Coefficient	Stiffness *K_c_* (N/mm)
Limb—Ground	150	20	2.2	0.58	0.46	20,000
