# Supplementary material for: Multibody Computer Model of the Entire Equine Forelimb Simulates Forces Causing Catastrophic Fractures of the Carpus during a Traditional Race
Source: Animals (Basel). 2022 Mar 16;12(6):737. doi: 10.3390/ani12060737 (PMC8944875; doi:10.3390/ani12060737)
Supplement: Supplementary file 1 [file animals-12-00737-s001.zip › Figure S1.pdf]

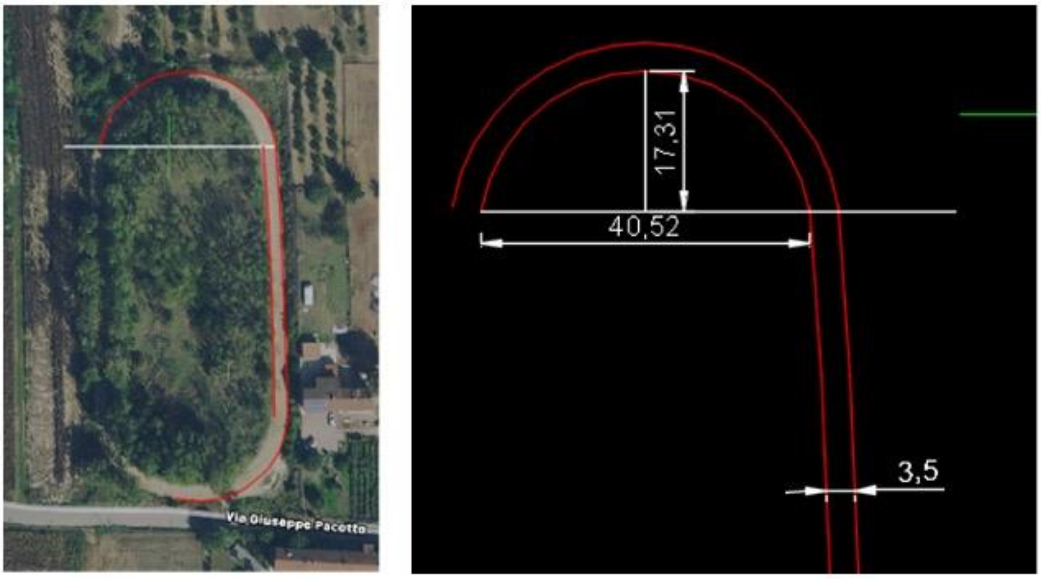

Figure S1. View from above and planimetry of the surrogate track used to simulate in vivo kinematic measurement during turn.
